# Efficacy of febuxostat on hyperuricemia and estimated glomerular filtration rate in patients with non-dialysis stage 3/4 chronic kidney disease and assessment of cardiac function: a 12-month interventional study

**DOI:** 10.3389/fneph.2025.1526182

**Published:** 2025-03-26

**Authors:** Yousuf Abdulkarim Waheed, Fan Yang, Jie Liu, Shifaa Almayahe, Karthick Kumaran Munisamy Selvam, Disheng Wang, Dong Sun

**Affiliations:** ^1^ Department of Nephrology, Affiliated Hospital of Xuzhou Medical University, Xuzhou, China; ^2^ Clinical Research Center for Kidney Disease, Xuzhou Medical University, Xuzhou, China; ^3^ Department of Nephrology, the Second Affiliated Hospital of Xuzhou Medical University, Xuzhou, China; ^4^ Medical College, University of Fallujah, Al Anbar, Iraq; ^5^ Department of Internal Medicine and Diagnostics, Xuzhou Medical University, Xuzhou, China

**Keywords:** febuxostat, hyperuricemia, chronic kidney disease, cardiovascular, troponin

## Abstract

**Objectives:**

Febuxostat, an oral medication for treating hyperuricemia (HUA), is a non-purine xanthine oxidase inhibitor that regulates serum uric acid (SUA) metabolism in patients with chronic kidney disease (CKD). However, the drug’s effectiveness in improving renal function in patients with non-dialysis stage 3/4 CKD remains unclear. Our aim is to investigate the efficacy of febuxostat on kidney function. In addition, the cardiac function will be assessed.

**Method:**

We conducted a single-center, interventional, randomized, controlled, open-label study. A total of 316 patients with non-dialysis stage 3/4 CKD, with SUA ≥6mg/dL in women and ≥7mg/dL in men, were assigned to either the febuxostat group (n=156) or the control group (n=160). The primary endpoint was the evaluation of changes in kidney biomarkers from baseline to 12 months of treatment, and any changes in cardiac biomarkers and echocardiographs were the secondary endpoint.

**Results:**

The primary endpoint was a comparison between the two groups from baseline to 12 months of treatment. SUA was significantly decreased in patients treated with febuxostat 40 mg (6.85 ± 0.41mg/dL at baseline and 5.27 ± 0.70mg/dL at 12 months of treatment, *P<0.001*) and this was associated with increased eGFR (34.48 ± 8.42ml/min at baseline and 38.46 ± 8.87ml/min at 12 months of treatment, *P<0.001*). There were significant decreases in high-sensitivity troponin T (19.50 ± 7.24ng/L at baseline and 16.98 ± 7.32ng/L at 12 months of treatment, *P<0.001)* and N-terminal-pro brain natriuretic peptide (941.35 ± 374.30pg/ml at baseline and 762.22 ± 303.32 pg/ml at 12 months of treatment, *P<0.001)* in the febuxostat group. These changes were also associated with increased left ventricular ejection fraction in the febuxostat group (50.47 ± 3.95% at baseline and 51.12 ± 3.96% at the end of the study, *P<0.001*).

**Conclusion:**

In the interventional group, febuxostat was well-tolerated and demonstrated a reduction in SUA associated with an increase in eGFR. This slowed down the progression of renal disease in patients with non-dialysis stage 3/4 CKD and improved cardiac function.

## Graphical Abstract

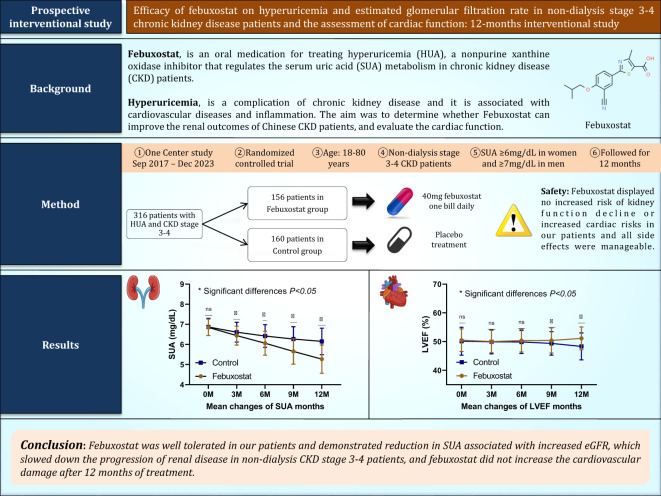

## Highlights

- Febuxostat reduced SUA in non-dialysis patients with stage 3/4 chronic kidney disease and was associated with an increase in eGFR levels after 12 months of treatment.
- Febuxostat alleviated the cardiac biomarkers and improved the left ventricular ejection fraction after 12 months of treatment.

## Introduction

The overproduction of uric acid (UA) in the body or reduced excretion of urate through the kidneys and gastrointestinal tract can lead to the development of hyperuricemia (HUA) (1). According to statistics, the prevalence of HUA in the Chinese adult population is approximately 14.0%, and it increases with the progression of chronic kidney disease (CKD) (2). HUA is associated with the risk of CKD progression in various situations, such as diabetic nephropathy and metabolic syndromes, and it is considered the second most common metabolic disorder in China (3, 4). HUA in CKD patients, if left untreated, accelerates the decline of kidney function and could cause end-stage kidney disease (ESKD). Some emerging evidence has also linked HUS to the development of cardiovascular disease (CVD) and increased mortality (5).

Dialysis patients have a high mortality rate and poor quality of life. Although renal transplantation is effective, its widespread application is limited by a lack of donors, the high cost of medical expenses, and the side effects of immunosuppressants. Therefore, it is necessary to find a way to control the acceleration of kidney function decline. Understanding and intervening in the risk factors related to HUA in the progression of CKD is a pivotal aspect of improving renal function in high-risk patients (6, 7). One of the first-line drugs for treating HUA in China is febuxostat (8). The drug is primarily metabolized in the liver, and no dose reduction is needed in patients suffering from renal dysfunction (9). Given this information, using febuxostat for therapeutic purposes could be effective and potent in lowering intracellular serum uric acid (SUA) due to its ability to block intracellular production accompanied by a decrease in extracellular levels.

However, the effects of febuxostat on HUS in non-dialysis stage 3/4 CKD are not fully clear, and its effects on cardiac function remain controversial and continue to spark debates. The objective of this study was to investigate the effect of febuxostat on HUA in patients with non-dialysis stage 3/4 CKD and to monitor overall renal function in general. Furthermore, we aimed to study and assess cardiac function with the anticipation of revealing novel insights into the prevention and treatment of HUA patients with CKD.

## Methods

### Study randomization and population

A single-center interventional, randomized, controlled, open-label study involving HUA patients with non-dialysis stage 3/4 CKD was conducted that aimed to evaluate the efficacy of febuxostat in regulating SUA over 12 months of observation. The study was conducted from September 2017 to December 2023 and participants were randomly allocated in a 1:1 ratio to the febuxostat group (n=156) or control group (n=160) using a computer-generated randomization sequence to ensure unbiased group assignment. After confirming eligibility, study investigators assigned participants to their respective groups. The open-label design meant that both participants and investigators were aware of treatment assignments, but laboratory parameters were analyzed using standardized, objective methods to mitigate measurement bias. The randomization protocol ensured balanced group sizes and minimized selection bias with no stratification. Inclusion criteria were as follows: [1] adults ≥ 18 years of age at the time of enrollment with CKD stage 3 or 4; [2] patients with HUA levels greater than or equal to SUA ≥6 mg/dL in women and ≥7 mg/dL in men and eGFR between 15 and 60 mL/min/1.73m², calculated according to the Chronic Kidney Disease Epidemiology Collaboration formula, CKD-EPI, which is based on serum creatinine levels, gender, and age at the time of enrollment (10, 29). An additional inclusion criterion was that patients should not be on any previous urate-lowering medication for at least 4 weeks before enrollment. Exclusion criteria were as follows: [1] patients who had severe liver disease; [2] dialysis patients, as dialysis treatment can alter the levels of SUA; [3] patients who received anti-hyperuricemic agents such as allopurinol and benzbromarone; [4] patients with a history of intolerance to febuxostat use; [5] patients with gout; [6] patients with a history of severe cardiovascular disease that required hospitalization within the previous 3-months; [7] patients receiving immunosuppressive therapy; [8] pregnant and lactating patients; [9] patients who were not eligible to participate in our trial. Physical examinations, clinical assessments, usage of medications, kidney parameters, cardiac parameters, and echocardiographs were all reviewed at the baseline of the study and 3, 6, 9, and 12 months (end of enrollment). Each investigator conducted the study in compliance with the local or regional regulatory requirements and with the ethical standards of the hospital. The study protocol was approved by the scientific research ethics committee at the Faculty of Nephrology, Xuzhou Medical University, ethics No. xyfy2017-kl043-01, and was registered on clinicaltrials.gov (ID: NCT03425708). All patients enrolled gave written informed consent at the time of enrollment.

### Study drug and administration

After providing informed consent, eligible patients were assigned to either the febuxostat group, which was administered a starting dose of 40 mg/d of febuxostat (11), or the control group receiving placebo treatment. All patients were provided with lifestyle guidance and changes to decrease SUA levels. Before treatment, the mean age, blood pressure, UA, and other related indicators and parameters were controlled to be approximately the same level in both groups *(P>0.05).* In the febuxostat group, patients were prescribed a daily dosage of 40 mg febuxostat starting from the time of enrollment (Brand: Unicom Febuxostat Tablets, Jiangsu Wang Bang Biochemical, and Pharmaceutical, Approval No.H20130058). Additionally, each group received lifestyle modifications and was treated with conventional conservative renal failure treatment (high-quality protein, low salt, low fat, and low phosphorus diet; blood pressure control; intestinal elimination of toxins; etc.). During the course of the trial, any adverse reactions were effectively managed and addressed by the responsible physicians. Patients with previously prescribed medications to control and manage their blood pressure or diabetes did not have their medications discontinued or altered throughout the duration of the trial. The medications included antihypertensive, antiplatelet, and diabetic agents. From the time of enrollment until the conclusion of the study, both groups were closely monitored. The initial treatment target was to decrease SUA levels and speed of eGFR change, along with treatment response. Other urate-lowering drugs were not allowed throughout the trial.

### Endpoints

The current research’s primary efficacy endpoint was the evaluation of the changes in kidney function biomarker levels from baseline to 12 months of treatment in patients receiving febuxostat 40mg, which include SUA, eGFR, serum creatinine (SCR), albumin (ALB), and blood urea nitrogen (BUN). The second efficacy endpoint was any alteration in cardiac enzymes, which include high-sensitivity troponin T (Hs-TnT), N-terminal-pro brain natriuretic peptide (NT_proBNP), creatinine kinase (CK), and creatinine kinase-MB (CK-MB), and echocardiographs such as left ventricular ejection fraction (LVEF), along with other variables that will be mentioned below. All laboratory assessments were carried out in the hospital’s laboratory and were collected at five specific time points. The individual responsible for conducting the laboratory tests remained blinded to information regarding the patients’ conditions and clinical records. An automated biochemical analyzer (Roche cobas8000, Roche, Switzerland) was utilized for the analysis of the samples. These endpoints will determine the efficacy and safety of using febuxostat in treating HUA in patients with non-dialysis stage 3/4 CKD.

### Assessment of echocardiographic parameters and variables

The echocardiographic findings were obtained from the participants’ charts and electronic records by our hospital specialists, who are experienced technicians using the standardized methods. The variables were collected and measured according to the American Society of Echocardiography guidelines (12, 13). By utilizing the bi-plane disk method, the LVEF was measured and calculated. The ventricular and arterial remodeling parameters, which include the left ventricular end-systolic dimension (LVESD), left ventricular end-diastolic dimension (LVEDD) and left ventricular posterior wall (LVPW), were also collected and measured according to the guidelines. The LVEDD was measured at end-diastole on parasternal views. The LVESD was measured from the parasternal long-axis view and at end-systole, and the LVPW was measured at end-diastole. All participants were followed for 12 months with the aim of performing five echocardiographic check-ups during the trial to assess the cardiac function. The baseline was defined as the patients’ first echocardiography.

### Statistical analysis

The study data were analyzed using SPSS 24.0 software on Windows 11. Q-Q plots were used to check whether the residuals were normally distributed. Normally distributed data were expressed as means ± standard deviation, and independent samples t-tests were utilized for comparisons between groups. Non-normally distributed data were expressed as medians and interquartile ranges. The enumeration data were expressed as percentages, and the Chi-squared test was used to compare groups. A mixed-effects repeated measures model was utilized to analyze the mean changes from baseline in kidney biomarkers and cardiac enzymes over 12 months. P*-*values <*0.05* were considered statistically significant. In addition, Graph Prism 8 was utilized to generate the figures.

## Results

### Baseline characteristics of patients

**Figure 1:**
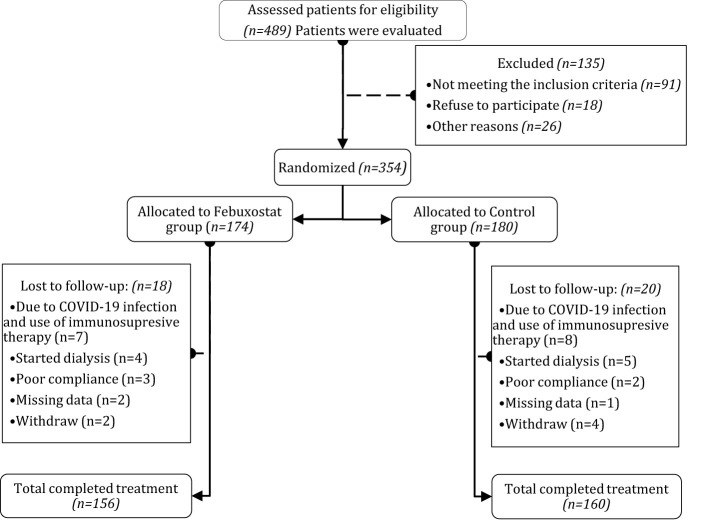
Flow chart of randomized controlled trial on the efficacy of febuxostat. After patients assessed for eligibility, patients were randomly distributed and assigned to either Febuxostat group or Control group, among the 489 patients, 354 patients were grouped and followed for 12 months. After exclusion, the total analyzed patients were Febuxostat group (n=156) and Control group (n=160). Patients with any sort of missing data were totally excluded from the study.

From September 2017 to March 2023, 354 patients were randomized into groups (febuxostat group = 174; control group = 180). Among the 354 participants, 316 patients (mean age 54.23 ± 13.36 years in the febuxostat group and 54.97 ± 13.29 years in the control group) completed the 12 months of follow-up (febuxostat group = 156; control group = 160) (**Figure 1**). The most common etiologies of CKD were chronic glomerulonephritis in 32% (febuxostat group = 46; control group = 48), hypertensive nephropathy in 28% (febuxostat group = 48; control group = 43), and diabetic nephropathy in 27% (febuxostat group = 41; control group = 47). Among our participants, 211 (66%) were diagnosed with stage 3a or 3b CKD, and 105 (33%) with stage 4 CKD. The initial demographic and clinical parameters of both groups showed no significant differences at baseline and many parameters, such as blood pressure, were well controlled *(P>0.05)*. The baseline characteristics of both groups and their coexisting diseases are listed in **Table 1**.

**Table 1:** Baseline analytical and characteristics in the study population.

Characteristic	All participants	Febuxostat	Control	*P Value*
No. of participants	*n=316*	*n=156*	*n=160*	
Demographic				
Age (Years)	54.88 ± 13.8	54.23 ± 13.36	54.97 ± 13.29	0.62
Male participants (%)	175 (55)	89 (57)	86 (53)	0.85
Female participants (%)	141 (44)	67 (42)	74 (46)	0.85
Etiology of chronic kidney disease				
Hypertension nephropathy (%)	91 (28)	48 (30)	43 (27)	0.79
Diabetic nephropathy (%)	88 (27)	41 (26)	47 (29)	0.78
Chronic glomerulonephritis (%)	94 (32)	46(33)	48 (31)	0.87
Nephrotic syndrome (%)	12 (3)	5(3)	7(4)	0.88
Other (%)	31(11)	16(10)	15(9)	0.95
Laboratory markers				
SUA(mg/dL)	6.86 ± 0.42	6.85 ± 0.41	6.87 ± 0.43	0.68
SUA – Male participants (mg/dL)	7.15 ± 0.29	7.11 ± 0.30	7.19 ± 0.28	0.59
SUA – Female participants (mg/dL)	6.50 ± 0.25	6.51 ± 0.27	6.49 ± 0.23	0.89
*eGFR (ml/min per 1.73m²)	34.16 ± 8.61	34.48 ± 8.42	33.84 ± 8.81	0.51
SCR (mg/dL)	2.51 ± 0.83	2.51 ± 0.85	2.51 ± 0.82	0.99
BUN (mmol/l)	16.11 ± 5.06	16.21 ± 5.54	16.02 ± 4.55	0.74
Alb (g/l)	35.49 ± 4.19	35.52 ± 3.57	35.46 ± 4.72	0.90
Hemoglobin (g/l)	116.61 ± 12.54	117.37 ± 13.14	115.88 ± 11.93	0.29
Hs-CRP (mg/l)	11.98 ± 4.90	12.06 ± 4.76	11.91 ± 5.04	0.78
Hs-TnT (ng/L)	19.20 ± 7.04	19.50 ± 7.24	18.91 ± 6.85	0.46
CK (u/l)	71.86 ± 11.04	72.24 ± 10.99	71.49 ± 11.11	0.54
CK-MB (ng/ml)	1.52 ± 0.44	1.55 ± 0.43	1.50 ± 0.46	0.34
NT_proBNP (pg/ml)	942.14 ± 346.09	941.21 ± 374.30	943.04 ± 317.37	0.96
TG (mmol/L)	2.69 ± 0.70	2.64 ± 0.66	2.75 ± 0.74	0.17
HDL-C (mmol/L)	1.87 ± 0.53	1.93 ± 0.57	1.81 ± 0.49	0.06
LDL-C (mmol/L)	3.39 ± 0.56	3.39 ± 0.63	3.40 ± 0.49	0.96
^a^Systolic BP (mmHg)	133.73 ± 10.12	134.38 ± 9.63	133.10 ± 10.57	0.26
^a^Diastolic BP (mmHg)	88.91 ± 5.27	89.31 ± 5.78	88.51 ± 4.71	0.17
^a^Echocardiography markers				
LVEF (%)	50.29 ± 4.42	50.47 ± 3.95	50.11 ± 4.84	0.46
LVEDD (mm)	55.62 ± 3.72	55.44 ± 4.04	55.80 ± 3.39	0.38
LVESD (mm)	34.77 ± 3.43	34.57 ± 3.52	34.96 ± 3.34	0.31
LVPW (mm)	9.79 ± 1.37	9.81 ± 1.41	9.77 ± 1.35	0.80
Medications				
Antiplatelet agent (%)	111 (35)	54 (34)	57 (35)	0.76
Diuretics (%)	109 (34)	53 (33)	56 (35)	0.78
ACEI/ARB (%)	164 (52)	81 (51)	83 (52)	0.86
β-blocker (%)	47 (14)	24 (15)	23 (14)	0.95
CCB (%)	39 (12)	20 (12)	19 (11)	0.96
Insulin (%)	93 (29)	45 (28)	48 (30)	0.87
Coexisting condition				
CKD stage 3a-3b (%)	211 (66)	106 (67)	105 (65)	0.85
CKD stage 4 (%)	105(33)	55 (35)	50 (31)	0.76
Hypertension (%)	196 (62)	97 (63)	99 (61)	0.93
Myocardial infarction (%)	33 (10)	17 (10)	16 (10)	0.98
Heart failure (%)	42 (13)	22 (14)	20 (12)	0.84
Diabetes mellitus (%)	90 (28)	46 (29)	44 (27)	0.86
Previous gout (%)	73 (23)	35 (22)	36 (22)	0.97
Previous cancer (%)	57 (18)	27 (17)	30 (18)	0.78
Smoking (%)	163 (51)	80 (50)	83 (52)	0.79
Alcohol use (%)	142 (45)	72 (45)	70 (44)	0.85

Variables are expressed as a mean ± SD or expressed as a percentage (%). No significant differences were observed between the different variables analyzed at baseline. Student’s t-test was utilized to compare the normally distributed data.

a Expressed as mean ± SD.

*eGFR (ml/min per 1.73m²) was calculated according to the Chronic Kidney Disease Epidemiology Collaboration formula.

^a^Variables were measured according to the American Society of Echocardiography guidelines.

SUA, serum uric acid; SCR, serum creatinine; BUN, blood urea nitrogen; eGFR, estimated glomerular filtration rate; Alb, albumin; Hs-CRP, high-sensitivity C-reactive protein; Hs-TnT, high-sensitivity troponin T; CK, Creatine kinase; CK-MB, Creatine kinase-MB; ACEI, angiotensin-converting enzyme inhibitor; ARB, angiotensin receptor blocker; CCB, calcium channel blocker; TG, triglyceride; HDL-C, high-density lipoprotein; LDL-C, low-density lipoprotein; LVEF, left ventricular ejection fraction; LVESD, left ventricular end-systolic dimension; LVEDD, left ventricular end-diastolic dimension; LVPW, left ventricular posterior wall.

### Effects of febuxostat on renal function

During the 12-month follow-up, six patients (1.8%) discontinued intervention (two in the febuxostat group; and four in the control group) for different reasons. The visual inspection of the Q-Q plot showed that SUA, eGFR, and other kidney markers were approximately normally distributed. Therefore, the Student’s t-test was utilized to measure the differences between groups.

**Figure 2:**
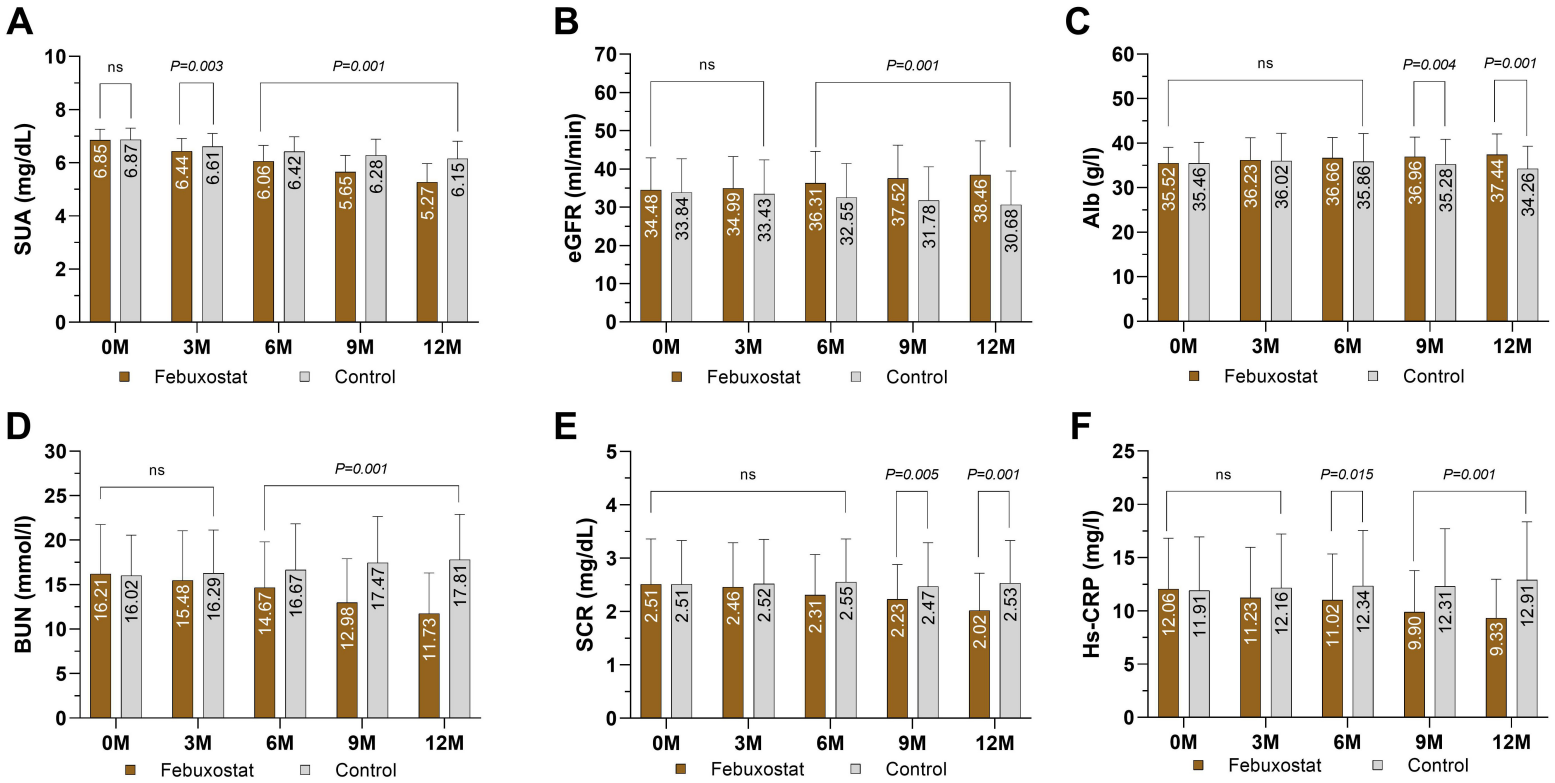
Mean changes of kidney markers and inflammation marker by treatment (febuxostat group n=156 vs. control group n=160) from baseline to 12 months of intervention. **(A)** serum uric acid, **(B)** estimated glomerular filtration rate, **(C)** albumin, **(D)** blood urea nitrogen, **(E)** serum creatinine, **(F)** High-sensitivity C reactive protein. (ns) not-significant. P<0.05 means statistical significant.

There was no significant difference in SUA between the febuxostat group and the control group at baseline [6.85 ± 0.41 and 6.87 ± 0.43 (95% CL, -1.01 to 0.66) mg/dL respectively, *P=0.685*]. The concentration of SUA decreased significantly in the febuxostat group compared to the control group at the 3-month follow-up [6.44 ± 0.47 (95% CL,-2.40 to 0.51) mg/dL, *P=0.003*]. This decreasing trend continued until the end of the trial. At the 12-month follow-up, there was a greater reduction in SUA in the febuxostat group compared to the control group [5.27 ± 0.70 (95% CL, -9.16 to -6.47) mg/dL, *P=0.001*] (**Table 2**, **Figure 2A**). These changes were associated with an increase in eGFR levels in the febuxostat group from baseline, [34.48 ± 8.42 (95% CL, -1.27 to 2.54) mL/min/1.73m², *P=0.515*] to the 12-month follow-up [38.46 ± 8.87 (95% CL, 5.83 to 9.73) mL/min/1.73 m² *P=0.001*] (**Table 2**, **Figure 2B**).

**Table 2:** Evolution of kidney and cardiac biomarker levels by treatment duration.

Variable	Baseline	3 months	6 months	9 months	12 months
SUA (mg/dL)					
Febuxostat	6.85 ± 0.41	¹6.44 ± 0.47	²6.06 ± 0.59	²5.65 ± 0.63	²5.27 ± 0.70
Control	6.87 ± 0.43	¹6.61 ± 0.49	²6.42 ± 0.56	²6.28 ± 0.61	²6.15 ± 0.66
eGFR (ml/min)					
Febuxostat	34.48 ± 8.42	34.99 ± 8.42	²36.31 ± 8.26	²37.52 ± 8.69	²38.46 ± 8.87
Control	33.84 ± 8.81	33.43 ± 8.94	²32.55 ± 8.92	²31.78 ± 8.80	²30.68 ± 8.77
BUN (mmol/l)					
Febuxostat	16.21 ± 5.54	15.48 ± 5.58	²14.67 ± 5.15	²12.98 ± 4.93	²11.73 ± 4.59
Control	16.02 ± 4.55	16.29 ± 4.86	²16.67 ± 5.19	²17.47 ± 5.19	²17.81 ± 5.09
SCR (mg/dL)					
Febuxostat	2.51 ± 0.85	2.46 ± 0.83	2.31 ± 0.76	¹2.23 ± 0.65	²2.02 ± 0.70
Control	2.51 ± 0.82	2.52 ± 0.83	2.55 ± 0.81	¹2.47 ± 0.82	²2.53 ± 0.80
Alb (g/l)					
Febuxostat	35.52 ± 3.57	36.23 ± 5.01	36.66 ± 4.61	¹36.96 ± 4.40	²37.44 ± 4.63
Control	35.46 ± 4.72	36.02 ± 6.18	35.86 ± 6.30	¹35.28 ± 5.61	²34.26 ± 5.08
Hs-CRP (mg/l)					
Febuxostat	12.06 ± 4.76	11.23 ± 4.74	²11.02 ± 4.32	²9.90 ± 3.87	²9.33 ± 3.63
Control	11.91 ± 5.04	12.16 ± 5.06	²12.34 ± 5.21	²12.31 ± 5.41	²12.91 ± 5.44
Hs-TnT (ng/L)					
Febuxostat	19.50 ± 7.24	18.93 ± 7.09	18.70 ± 6.53	²16.94 ± 7.34	²16.98 ± 7.32
Control	18.91 ± 6.85	19.41 ± 6.58	20.73 ± 6.98	²22.94 ± 7.75	²23.90 ± 7.10
CK (u/l)					
Febuxostat	72.24 ± 10.99	69.97 ± 12.53	²70.67 ± 14.80	²69.51 ± 14.21	²67.46 ± 14.36
Control	71.49 ± 11.11	71.57 ± 12.53	²77.13 ± 13.46	²78.75 ± 14.65	²86.21 ± 13.97
CK-MB (ng/ml)					
Febuxostat	1.55 ± 0.43	1.45 ± 0.45	¹1.39 ± 0.48	²1.37 ± 0.47	²1.30 ± 0.47
Control	1.50 ± 0.46	1.48 ± 0.43	¹1.56 ± 0.50	²1.73 ± 0.45	²1.86 ± 0.40
NT_proBNP (pg/ml)					
Febuxostat	941.21 ± 374.30	904.35 ± 338.78	874.08 ± 304.18	800.36 ± 306.65	²762.22 ± 303.32
Control	943.04 ± 317.37	925.14 ± 295.62	908.44 ± 319.58	887.43 ± 269.81	²875.88 ± 309.39
LVEF (%)					
Febuxostat	50.47 ± 3.95	50.02 ± 3.92	50.32 ± 3.95	¹50.39 ± 4.38	²51.12 ± 3.96
Control	50.11 ± 4.84	49.91 ± 4.22	49.85 ± 4.00	¹49.36 ± 4.10	²48.31 ± 4.69
LVEDD (mm)					
Febuxostat	55.44 ± 4.04	53.49 ± 4.38	¹53.40 ± 4.28	²53.60 ± 3.59	²53.79 ± 3.84
Control	55.80 ± 3.39	52.96 ± 3.04	¹52.41 ± 3.03	²51.69 ± 2.85	²51.47 ± 2.80
LVESD (mm)					
Febuxostat	34.57 ± 3.52	33.92 ± 3.65	¹33.46 ± 3.43	²33.11 ± 3.62	²32.82 ± 3.47
Control	34.96 ± 3.34	34.50 ± 3.21	¹34.43 ± 2.62	²34.88 ± 2.56	²35.18 ± 2.54
LVPW (mm)					
Febuxostat	9.81 ± 1.41	9.73 ± 1.45	9.39 ± 1.69	9.79 ± 1.24	^a^9.76 ± 1.37
Control	9.77 ± 1.35	9.98 ± 1.52	9.56 ± 1.87	10.01 ± 1.41	^a^10.06 ± 1.38

Measurement data are expressed as mean ± standard deviation; the endpoint of the study was compared with that before treatment.

Student’s t-test was used to compare the variables; *P<0.05* is considered statistically significant.

SUA, serum uric acid; SCR, serum creatinine; BUN, blood urea nitrogen; eGFR, estimated glomerular filtration rate; Alb, albumin; Hs-CRP, high-sensitivity C-reactive protein; Hs-TnT, high-sensitivity troponin T; CK, Creatine kinase; CK-MB, Creatine kinase-MB; LVEF, left ventricular ejection fraction; LVESD, left ventricular end-systolic dimension; LVEDD, left ventricular end-diastolic dimension; LVPW, left ventricular posterior wall.

¹ Significant differences between the groups *(P<0.005).*

² Significant differences between the groups *(P<0.001).*

^a^ Significant differences between the groups *(P<0.059).*

ALB levels showed no significant difference between groups at baseline [35.52 ± 3.57 and 35.46 ± 4.72 (95% CL, -0.87 to 0.98) g/l, respectively, *P=0.904*]. At the 12-month follow-up, ALB showed a significant increase in the febuxostat group compared to the control group [37.44 ± 4.63 (95% CL, 2.10 to 4.25) g/l, *P=0.001*] (**Table 2**, **Figure 2C**). There was no significant difference observed between groups in the BUN level at baseline [16.21 ± 5.54 and 16.02 ± 4.55 (95% CL, 0.93 to 1.31) mmol/l, respectively, *P=0.743*]. At the 12-month follow-up, the BUN level had significantly decreased in the febuxostat group [11.73 ± 4.59 (95% CL, -7.16 to -5.01) mmol/l, *P=0.001*] (**Table 2**, **Figure 2D**). In addition, SCR showed no significant difference at baseline between both groups (2.51 ± 0.85 and 2.51 ± 0.82 [95% CL, -0.18 to 0.15] mg/dL, respectively, *P=0.997*). At the 12-month follow-up, SCR significantly decreased with febuxostat with a mean change (2.02 ± 0.70 [95% CL, -5.98 to -3.01] mg/dL, *P=0.001*) (**Table 2**, **Figure 2E**).

The eGFR slope serves as a biologically plausible surrogate marker of CKD progression. To compare the eGFR slope between groups, a linear mixed-effects model was employed. The control group demonstrated a significant decline in eGFR of -0.26 mL/min/1.73m² per month [95% CI: -0.30 to -0.22; *P<0.001*]. In contrast, the febuxostat intervention group showed a positive eGFR slope of +0.35 mL/min/1.73m² per month, which was calculated as the control slope of -0.26 + interaction effect of 0.62; *P< 0.001* for the interaction term. This interaction effect between febuxostat and time of +0.62 mL/min/1.73m² per month (95% CI: 0.55 to 0.66; *P< 0.001*) indicates that febuxostat significantly attenuated eGFR decline compared to the control group. While the baseline eGFR did not differ between groups (febuxostat vs. control: +0.20 mL/min/1.73m²; 95% CI: -1.69 to 2.09; *P< 0.837*), the divergence over time suggests a therapeutic benefit of febuxostat in preserving eGFR decline (**Table 3**).

**Table 3:** Linear mixed-effects model for eGFR over time in both groups.

Fixed effects	Estimated (95% CL)	SE	*P Value*
Intercept (baseline eGFR)	34.06 (32.73 to 35.38)	0.67	0.001
Febuxostat vs. control (baseline)	0.18 (-1.70 to 2.07)	0.96	0.848
Time (control group)	-0.26 (-0.30 to -0.22)	0.01	0.001
Time (febuxostat group)	0.61 (0.55 to 0.66)	0.02	0.001

Patients receiving febuxostat demonstrated a significant improvement in eGFR over time, whereas the control group experienced a progressive decline. Slopes are presented per month.

eGFR, estimated glomerular filtration rate; CL, confidence interval; SE, stranded error.

### Effect of febuxostat on inflammatory markers

At baseline, the level of the inflammatory marker Hs-CRP was 12.06 ± 4.76 ng/L in the febuxostat group and 11.91 ± 5.04 ng/L in the control group, with no statistical significance (*P=0.788)*. At the 3-month follow-up, there was a significant decrease in the febuxostat group (-1.04) whereas the control group showed an increase of +0.43, [11.02 ± 4.76 and 12.34 ± 5.21 (95% CL, -3.44 to -2.35) ng/L, respectively, *P=0.015*]. Similarly, at the 12-month follow-up, Hs-CRP continued to decrease, whereas the control group showed an increase [9.33 ± 3.63 and 12.91 ± 5.44 (95% CL, -4.60 to -2.54) ng/L, respectively, *P=0.001*] (**Table 2**, **Figure 2F**).

### Effect of febuxostat on cardiac biomarkers

A reduction in renal function was associated with increased levels of cardiac biomarkers; however, an improvement in eGFR and renal function was associated with a decrease in cardiac biomarkers. It is important to mention that the patients with preexisting cardiovascular disease were already taking medications to control blood pressure and CVD. In total, 196 (62%) patients had hypertension, 33 (10%) had a previous myocardial infarction, and 42 (13%) had heart failure.

**Figure 3:**
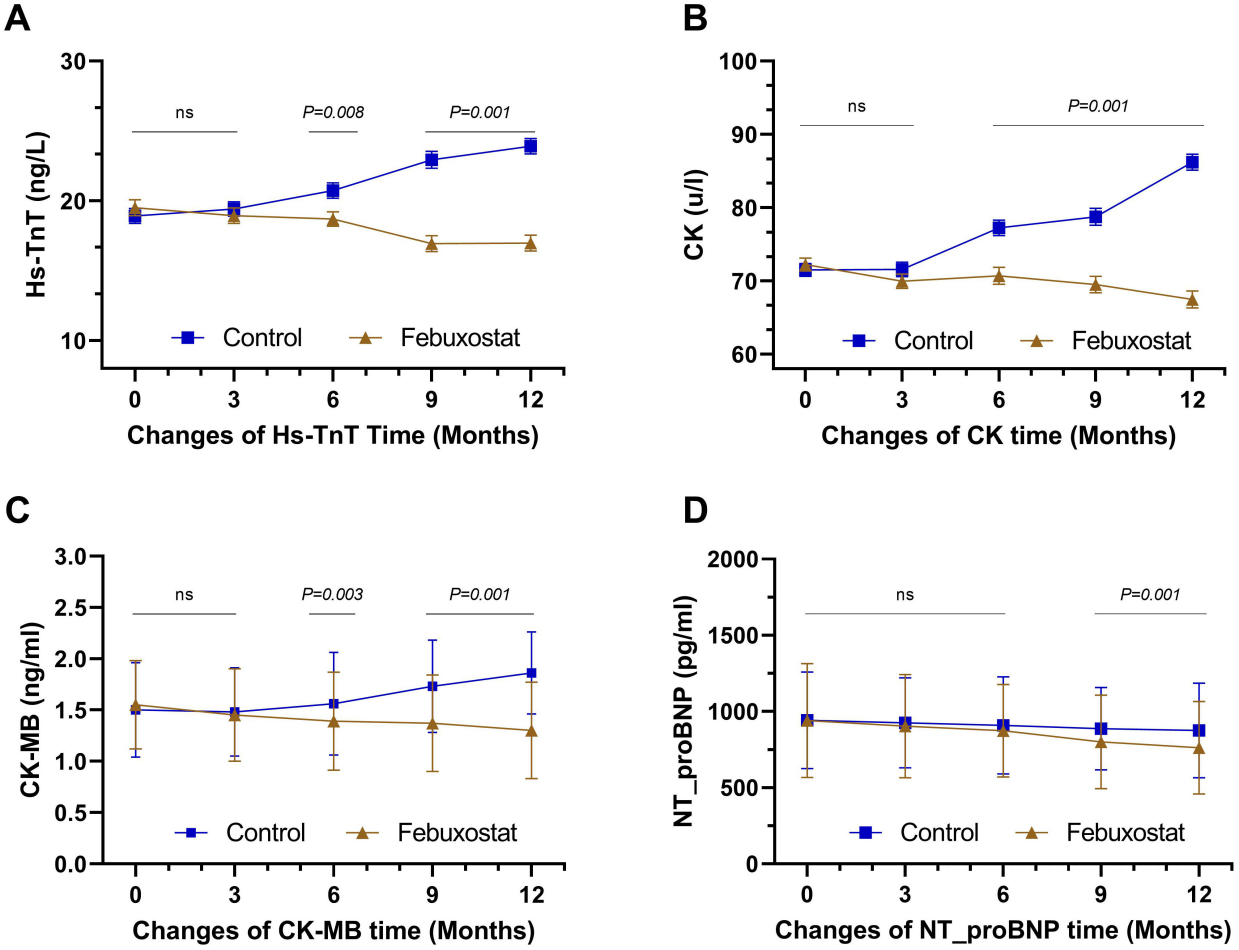
Mean changes of cardiac biomarkers over time by treatment (febuxostat group n=156 vs. control group n=160) from baseline to 12 months of intervention. **(A)** high-sensitivity troponin T, **(B)** creatine kinase, **(C)** creatine kinase-MB, **(D)** N-terminal b-type natriurtic peptide. (ns) not-significant. P<0.05 means statistical significant.

At baseline, Hs-TnT levels in the febuxostat and control groups were controlled at approximately the same level [19.50 ± 7.24and 18.91 ± 6.85 (95% CL, -0.97 to 2.14) ng/L, respectively, *P=0.462*]. At the 12-month follow-up, there was a significant decrease in the febuxostat group compared to the control group, which had an increased level [16.98 ± 7.32 and 23.90 ± 7.10 (95% CL, -8.50 to -5.31) ng/L, respectively, *P=0.001*] (**Table 2**, **Figure 3A**). Similarly, CK and CK-MB levels demonstrated similar results; there was a significant decrease in CK in the febuxostat group compared to the control group [67.46 ± 14.36 and 86.21 ± 13.97 (95% CL, -21.88 to – 15.61) u/l, respectively, *P=0.001*] (**Table 2**, **Figure 3B**) and CK-MB at the 12-month follow-up [1.30 ± 0.47 and 1.86 ± 0.40 (95% CL, -0.65 to – 0.46) ng/ml, respectively, *P=0.001*] (**Table 2**, **Figure 3C**).

At baseline, there was no significant difference in the level of NT_proBNP between the febuxostat group and the control group [941.21 ± 374.30 and 943.04 ± 317.37 (95% CL, -78.58 to 74.90)pg/ml, respectively, *P=0.962*]. The 9-month and the 12-month follow-ups showed significant decreases between groups with 800.36 ± 306.65 and 887.43 pg/ml (*P=0.011*), and 762.22 ± 303.32 and 875.88 ± 309.39 pg/ml (*P=0.001)*, respectively (**Table 2**, **Figure 3D**). Although the level of NT_proBNP decreased in both groups, the mean decrease in the febuxostat group was greater than that in the control group.

### Effects of febuxostat on the echocardiographs

To further evaluate cardiac function, echocardiographic findings were collected and compared throughout the trial five times from baseline to 12 months of treatment (end of the study). Any participant who failed to perform an echocardiographic examination was excluded from the trial.

**Figure 4:**
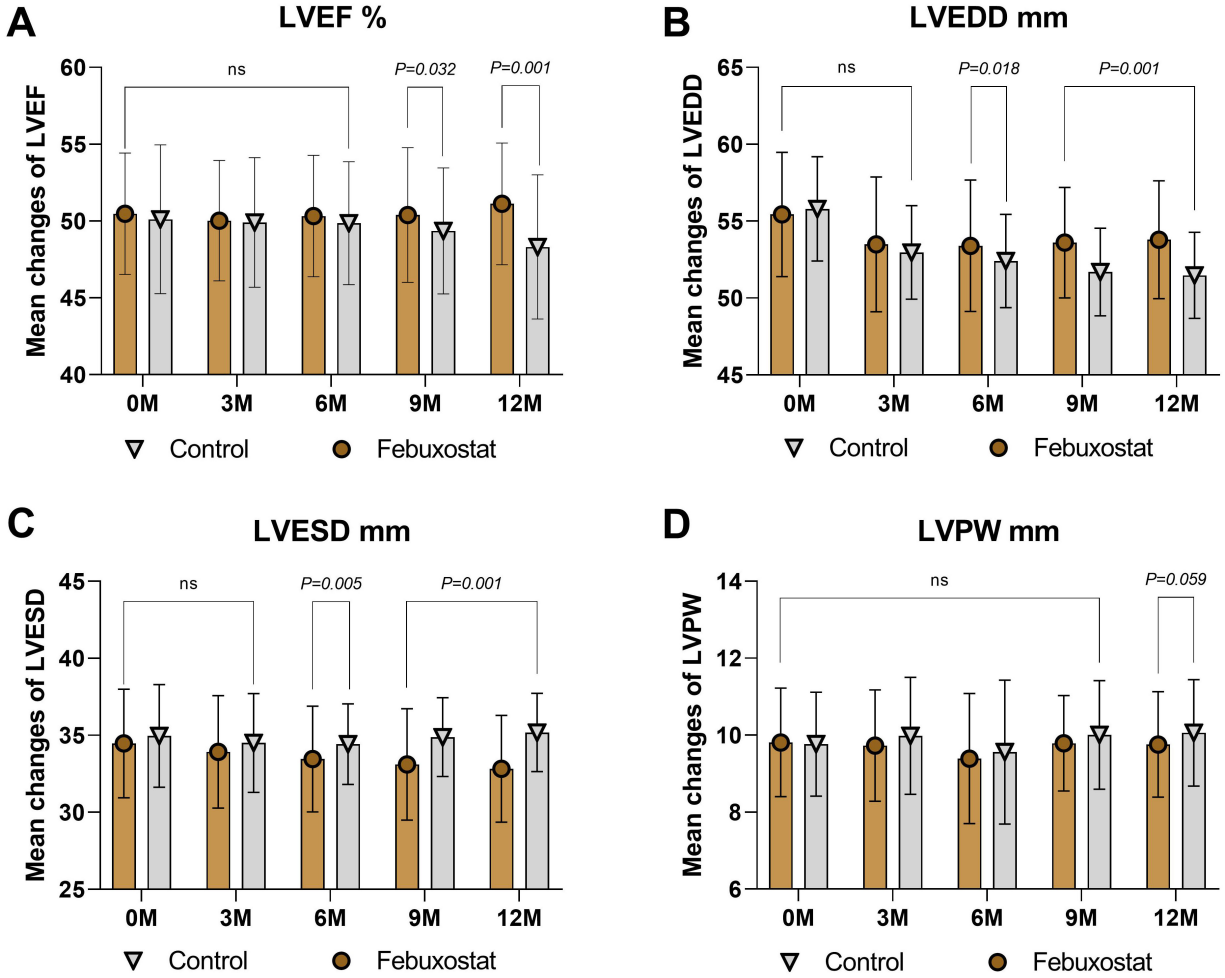
Mean changes in the echocardiograph findings by treatment (febuxostat group n=156 vs. control group n=160) from baseline to 12 months. **(A)** left ventricular ejection fraction, **(B)** left ventricular end-diastolic dimension, **(C)** left ventricular end-systolic dimension, **(D)** left ventricular posterior wall. (ns) not-significant. P<0.05 means statistical significant.

There was no significant difference in LVEF between the febuxostat group and the control group [50.47 ± 3.95% and 50.11 ± 4.84% (95% CL, -0.61 to 1.34), respectively, *P=0.461*]. At the 12-month follow-up, LVEF showed a significant increase in the febuxostat group compared to the control group [51.12 ± 3.96% (95% CL, 1.84 to 3.77), *P=0.001*] (**Table 2**, **Figure 4A**). At baseline, the level of LVEDD showed no significant difference between groups [55.44 ± 4.04% and 55.80 ± 3.39% (95% CL, -1.18 to 0.46), respectively, *P=0.386*]. At the 6-, and 9-, 12- month follow-ups, there was a significant decrease in the febuxostat group compared to the control group. The 12- month mean values [53.79 ± 3.84% and 51.47 ± 2.80% (95% CL, 1.58 to 3.07), respectively, *P*=0.001] (**Table 2**, **Figure 4B**). At baseline, the value of LVESD showed no statistical difference between both groups (34.57 ± 3.52 and 34.96 ± 3.34 [95% CL, -1.14 to 0.37] % respectively, *P=0.319*). The level of LVESD showed a significant decrease in the febuxostat group at the 6-, 9-, and 12- month follow-ups compared to the control group. The 12- month mean values [32.82 ± 3.47% and 35.18 ± 2.54% (95% CL, -3.03 to -1.68), respectively, *P*=0.001] (**Table 2**, **Figure 4C**).

Interestingly, the level of LVPW showed a significant difference after 12 months of treatment between groups [baseline = 9.81 ± 1.41% and 9.77 ± 1.35% (95% CL, -0.26 to 0.34), respectively, *P=0.802*; 12 months = 9.76 ± 1.37% and 10.06 ± 1.38% (95% CL, -0.59 to 0.01), respectively, *P=0.059*] (**Table 2**, **Figure 4D**). Based on these findings, we confirm that febuxostat has no adverse reactions on the echocardiographs and may lead to improved cardiac function.

### Adverse reactions

Febuxostat displayed no increased risk of kidney disease progression or increased cardiac risk in the febuxostat group, and all side effects were manageable. During the study follow-up period, three patients (0.9%) developed hypertension after 3, 8, and 9 months of treatment: two (1.2%) in the febuxostat group and one (0.6%) in the control group. The three patients were older adults, and during the trial, their side effects were managed by experts, and there were no further side effects. Hs-CRP levels in the febuxostat group remained stable and decreased throughout the study, whereas they showed a significant increase in the control group. In total, 23 (7.2%) cases of mild to moderate infections such as upper respiratory infections and urinary tract infections were identified during the trial, 10 (6.4%) in the febuxostat group and 13 (8.1%) in the control group. These were resolved with antibiotic treatment administered by experts, and no cases were discontinued. A few patients experienced vomiting and/or diarrhea; however, these symptoms vanished after a few days. In general, there were no obvious unfavorable events in the two groups.

## Discussion

Our study was mainly designed to evaluate the efficacy of febuxostat in treating HUA in patients with stage 3/4 CKD and to assess cardiac function. It is important to note that it did not aim to compare the drug efficacy with other UA-lowering agents. In this 12-month intervention, febuxostat was able to reduce SUA significantly, and this reduction was associated with an increase in eGFR levels of +3.98 ml/min per 1.73m² in the febuxostat group when compared to the control group, which showed a substantial decrease of -3.16 ml/min per 1.73m² throughout the study. Furthermore, febuxostat improved cardiac function by lowering cardiac biomarkers and increasing LVEF by +0.65% compared to the control group, which showed increased cardiac biomarkers and decreased LVEF by -1.8%.

HUA may induce renal afferent arteriopathy, which leads to increased glomerular hydrostatic pressure and thus leads to kidney function decline. Since there was a reduction in SUA in both groups, the febuxostat group still had a significantly reduced SUA and UA metabolism in patients with non-dialysis stage 3-4 CKD, adding to the improvement in eGFR and kidney function in the same group. We suggest that the reduction in SUA levels and increase in eGFR were associated with febuxostat use (14). The renoprotective effects of febuxostat in patients with CKD have been reported in multiple studies. Hsu et al. (15) conducted a 13-year cohort study and reported that febuxostat can contribute to lowering SUA levels and reduce the risk of ESKD in CKD populations. Our study results were also consistent with these findings, as the number of hospitalized patients was reduced in the febuxostat group. Increased eGFR levels in HUA patients have been previously researched. Whelton et al. (16) conducted a study in patients using febuxostat and found a significant increase in eGFR levels as SUA decreased; eGFR improved by +1 ml/min per 1.73m² for every -1 mg/dL decrease in SUA. These findings were in non-CKD gouty populations. In our CKD population, increased levels of eGFR were observed with each reduction in SUA levels throughout the study period. Febuxostat is safe to administer in patients with impaired renal function due to its clearance, which is mediated by hepatic metabolism, unlike other UA-lowering agents that are mainly excreted in the kidneys (17). In addition, the febuxostat group demonstrated a positive eGFR slope of 0.35 mL/min per month vs. -0.26 mL/min per month in the control group (*P<0.001)* (**Table 3**). These findings further suggest the potential renoprotective effects of febuxostat in patients with CKD. However, the FEATHER trial reported that febuxostat had no significant benefit in reducing the eGFR slope (17). This may be explained by the differences in study populations and baseline CKD severity.

A reduction in the levels of SCR was also observed in the febuxostat group at -0.49 mg/dL after 12 months of treatment compared to the control group, which demonstrated a substantial increase. This finding strengthened the effect of febuxostat in preventing the progression of renal function. Although the population and criteria of the aforementioned studies are different, taking our findings into account, we can conclude that febuxostat is a promising drug for treating HUA in patients with stage 3/4 CKD, and it can improve the quality of life of these patients with no side effects on their renal function.

Our study shows that febuxostat may confer renoprotective benefits, in contrast to a neutral effect reported in the CKD-FIX trial with allopurinol (30). This discrepancy may be suggestive of differences in the underlying mechanisms between the two drugs. As a non-purine xanthine oxidase inhibitor, febuxostat may exert a much broader and more dependable reduction in urate levels in patients with CKD, especially at higher doses, where allopurinol is often limited due to safety concerns. Therefore, the multifaceted features of febuxostat, such as its protective effects against oxidative stress and inhibition of the NLRP3 inflammasome, may provide renal protection beyond urate lowering, an effect that cannot be ascribed to allopurinol. However, differences in trial populations, stages of CKD, comorbidities, study designs, dosing regimens, and follow-up duration may have also contributed to these divergent results. Moreover, our findings align with the mechanistic and clinical framework proposed by Cicero et al. (31), which summarized evidence on the renoprotective potential of febuxostat in CKD. The review highlights febuxostat’s unique dual ability to lower SUA and suppress xanthine oxidase-driven oxidative stress, a pathway implicated in renal inflammation and fibrosis. Our observation that febuxostat improved eGFR supports their hypothesis, potentially through antioxidant and anti-inflammatory mechanisms.

Another key finding of our study is that, after 12 months of treatment with febuxostat, the Hs-TnT level significantly decreased by -2.52 ng/L compared to the control group, which showed a substantial increase of +4.99 ng/L. The interpretation of increased levels of Hs-TnT in patients with CKD is challenging, especially when suspecting acute myocardial infarction (AMI). Renally impaired patients are known to exhibit an elevated risk of CVD with atypical symptoms (18). Previous studies revealed that of patients with eGFR <45 mL/min/1.73 m² and no history of AMI, approximately 65% have increased levels of Hs-TnT (19). This increase in Hs-TnT in patients with CKD has been linked to increased myocardial stress or troponin retention (20). SUA is considered to be a CVD predictor because oxidative stress and urate crystals can induce an inflammatory reaction that can contribute to the development of atherosclerosis, which means that regulating SUA can reduce the incidence of cardiovascular damage (21, 22). At the 12-month follow-up, we could clearly see that the decreased level of cardiac markers in the febuxostat group was associated with a reduction of SUA level and an increase in eGFR levels. Previous clinical data strongly suggest a correlation between changes in Hs-TnT and renal function (23). Interestingly, our results revealed a significant reduction in the level of NT_proBNP in the febuxostat group compared to the control group at the end of the study. Previous studies have demonstrated a strong correlation between renal dysfunction and NT_proBNP levels (24). Hence, renal and cardiac function can be assessed together according to the level of influence of NT-proBNP concentrations. Therefore, we speculate that the prognosis of cardiac biomarkers may be due to the association with renal function. An improvement in eGFR levels was observed in our study, which may have led to an improvement in cardiac biomarkers.

Another key finding of our study is that febuxostat treatment improved the cardiac function findings on echocardiography compared to the control group (**Figure 4**). Increased levels of SUA have been found to inhibit the production of nitric oxide by vascular endothelial cells and impede their growth and movement (25). Previous observational studies and meta-analyses have linked high SUA levels to poor cardiac function, increased mortality, and reduced exercise capacity (26, 27). Elevated SUA levels may contribute to abnormal echocardiographic results by impacting endothelial function and causing inflammation. Therefore, SUA may serve not only as a prognostic marker for CVD but also as a potential target for interventions aimed at improving cardiac function. Several researchers have demonstrated that treating elevated SUA levels can lead to improved LVEF (28). Our results showed an increase in LVEF by +0.65% in the febuxostat group, the same group that showed good results in the reduction of SUA levels and improvement in cardiac function. Akihiro Nakagomi et al. (28) revealed that febuxostat is effective in reducing SUA levels and can improve LVEF. These results corroborate our findings.

Federica et al. (32) demonstrated that women are underrepresented in controlled clinical trials testing SUA-lowering drugs. In our study, women constituted 44% of the enrolled participants (n=144/316), reflecting a lower enrollment rate compared to men. This stems from our sex-specific SUA inclusion thresholds (≥6mg/dL for women vs. ≥7mg/dL for men), which may have disproportionately enrolled women with comparatively lower SUA elevations. While our trial was not powered to detect sex-specific differences, the underrepresentation of women limits generalizability to this high-risk subgroup, underscoring the need for sex-stratified analyses in future trials. Addressing this gap is important, as hormonal variations may alter febuxostat’s efficacy or safety profile in women, particularly in late-stage CKD.

Despite these findings, our study has several limitations that may influence the interpretation of our results. The sample was relatively small and the follow-up time was relatively short. In addition, it was a single-center study. Another key limitation is the lack of urinary albumin-to-creatinine ratio (ACR) and protein-to-creatinine ratio (PCR) measurements. These biomarkers are essential for detecting early kidney dysfunction and quantifying proteinuria with high sensitivity. Their absence may result in an underestimation of renal impairment. Moreover, we did not assess patient-centered outcomes such as symptom burden or hospitalizations for heart failure or fluid overload, which are critical indicators of clinical relevance in CKD populations. The absence of these data limits our ability to determine whether the observed benefits of febuxostat translate into meaningful improvements in patients’ quality of life. Finally, we did not include patients taking different doses of febuxostat to study the effective dose of the drug. Future studies should incorporate these endpoints to confirm whether the observed improvements in kidney function translate into tangible clinical benefits. In conclusion, the current study showed that febuxostat has good lowering effects on SUA in non-dialysis stage 3/4 CKD and was associated with an increased eGFR levels and improved renal function. Furthermore, cardiac biomarkers decreased in patients taking febuxostat and this reduction may improve echocardiographic findings, which may improve the systolic function, LVEF, and quality of life in patients.

## Data Availability

The raw data supporting the conclusions of this article will be made available by the authors, without undue reservation.
